# Highlighting the burden of malarial infection and disease in the neonatal period: making sense of different concepts

**DOI:** 10.1186/s12936-020-03394-3

**Published:** 2020-08-28

**Authors:** Abel Nhama, Rosauro Varo, Quique Bassat

**Affiliations:** 1 Centro de Investigação em Saúde de Manhiça (CISM), Maputo, Mozambique; 2 Instituto Nacional de Saúde (INS), Maputo, Mozambique; 3 ISGlobal, Hospital Clínic, Universitat de Barcelona, Barcelona, Spain; 4 ICREA, Pg. Lluís Companys 23, Barcelona, 08010 Spain; 5 Pediatric Infectious Diseases Unit, Pediatrics Department, Hospital Sant Joan de Déu (University of Barcelona), Barcelona, Spain; 6 Consorcio de Investigación Biomédica en Red de Epidemiología y Salud Pública (CIBERESP), Madrid, Spain; 7 Barcelona Institute for Global Health, Carrer Roselló 132, Barcelona, Sobreátic 08036 Spain

## Background

In malaria-endemic areas, the first year of life is indisputably considered one of the most vulnerable periods for malaria disease, and malaria-associated mortality in this age group is typically high, usually as a consequence of severe anaemia, among other possible complications [1]. However, malaria disease during the first 4 weeks of life (which includes both congenital and neonatal malaria) is comparatively much less common than among older infants. Congenital and neonatal malaria show important differences in terms of acquisition route and timing of the infection. They both are also importantly influenced by background malaria endemicity and degrees of population-acquired immunity.

## Acquisition route

Malaria in the first weeks of life can essentially arise from two very distinct transmission mechanisms. It can either be vertically transmitted from an infected pregnant mother to the child (in utero or during delivery) [2], or it can be acquired through a mosquito bite transmitting the infective forms of the parasite (sporozoite). In the first case, it is often challenging to determine whether the infection is derived from parasites acquired before delivery or as a result of contamination by maternal blood at birth. In the second case, and given the necessary incubation period of the parasite (~ 10–14 days, species dependant), it would be difficult to conceive any newborn becoming sick during its first week of life through this transmission route. By convention, therefore, it has been agreed to use different nomenclature to differentiate the assumed transmission route. Malaria cases detected in the first 7 days of life are called congenital malaria, whereas those detected henceforth and until 28 days of life are termed neonatal malaria. Such a distinction between congenital and neonatal malaria is relevant in endemic areas, because prevention methods designed to avoid infection in the baby should differentially target the pregnant mother or the baby. Outside of malaria endemic areas, malaria in the newborn is always assumed to have been transmitted congenitally, irrespective of the time of onset of symptoms.

### The importance of the background immunity status of the population against malaria

One of the fundamental factors in the current understanding of the pathophysiology and clinical presentation of malaria is related to the presence (or not) of any degree of acquired immunity to malaria from the human host [3]. Indeed, in malaria-endemic areas, repeated exposure to infectious mosquito bites leads to the progressive development of a natural immune response that will eventually protect children and adults from the deleterious consequences of malaria disease (including death). However, such acquired immunity is not sterilizing, and therefore, infections may still occur throughout life, even though they are not necessarily accompanied by clinical disease, a phenomenon known as malaria tolerance [4]. Thus, in malaria-endemic settings with moderate to high transmission, it is very common to find malaria parasites among symptomless individuals, and such infections may not necessarily require treatment as they can be considered incidental findings. This is radically different to what occurs in settings where malaria is less common or where autochthonous transmission does not occur, as immunity will not have developed, and all individuals remain equally naïve and susceptible to the disease once they get infected, irrespective of their age. Newborns could also be considered similarly naïve and susceptible to malaria, although they may passively acquire protecting antibodies from their mothers during pregnancy and/or breastfeeding.

### The perceived scarce malaria burden during the neonatal period

As a demonstrative example, an analysis of morbidity surveillance data from outpatients attending a Mozambican district hospital in a high transmission setting, and for which all patients with fever were systematically screened for malaria, signalled that only 2.7% of all newborns seen (~ 2000) had a clinical diagnosis of malaria, whereas this percentage increased to up to 40% among older children [5]. Several factors could explain why malaria during the neonatal period has been traditionally considered a rare phenomenon in malaria-endemic areas. As previously mentioned, in these settings, the passive transfer of antibodies from pregnant mothers, which generally start to wane after the first 3 months of life would explain why clinical malaria is uncommon before that period. Fetal haemoglobin (HbF), whose presence inside the young infant’s erythrocytes is known to hinder parasite growth, may also contribute to reduced vulnerability in the newborn to malaria, during the time it can still be detected in circulation [6]. Finally, the “sheltering” conferred during the first month of life to the many layers of clothing that newborns usually are dressed with, together with the closeness to the mother (for instance sleeping together under the same bed net), cannot be disregarded, as additional factors of protection. Of these arguments, the first two also explain why congenital malaria is often clinically silent and why potential symptoms among those cases are either absent, or rather unspecific (including fever or hypothermia, jaundice, hepato-splenomegaly, poor feeding) and often mild in nature. However, there is an evident discrepancy between clinical malaria cases in this period (relatively infrequent), and the prevalence of malaria infections (much more common), when proactively investigated. Indeed, when using more sensitive diagnostic methods, congenital infections seem to occur quite frequently among neonates born of women experiencing malaria during pregnancy, as confirmed by the high prevalence (up to 32% in some series) of PCR-detected malarial infection in cord blood [7].

### Clinical malaria among newborns of naïve to malaria pregnant mothers

The aforementioned scenario from malaria-endemic areas cannot be extrapolated to babies born of malaria-naïve–or with little acquired malaria immunity–pregnant women, whereby congenital transmission usually leads to a clinically overt and life-threatening episode. This is the situation of women infected with malaria in low-endemic settings, or as travellers. Given this, it is no surprise that the literature on congenital malaria is very much biased towards case-reports/series from very low- or non-endemic areas [8–13], whereby the differential diagnosis of the sick newborn typically requires excluding bacterial sepsis. Needless to say, these are often medical emergencies, with the added complexity of being tortuous to diagnose when expertise in malaria is missing and malaria is not initially considered as part of the differential diagnosis.

### A new approximation to the real burden of congenital and neonatal malaria

Celestin Danwang and colleagues [14] have conducted a systematic review and meta-analysis of 22 observational studies reporting on congenital (0–7 days) and neonatal (0–28 days) clinical malaria, including a total of 28,083 neonates from 14 malaria-endemic countries, in order to assess its pooled prevalence in this specific age group. Importantly, their results suggest a prevalence of congenital malaria of 40.4/1000, and a prevalence of neonatal malaria of 12/1000, with no apparent differences between point estimates from pooled studies from sub-Saharan Africa, and studies from other continents, although the limited examined data from the latter forcefully limit the generalizability of their conclusions. Such results complement a similar and recently published meta-analysis [15], restricted to congenital malaria only, and assessing 8148 newborns, who postulated an overall global prevalence of 6.9%, with a high inter-country variability (with estimates as high as 46.7% in Nigeria). Interestingly, the authors also confirmed congenital malaria to be more frequent in settings with unstable malaria transmission, a finding in line with the hypothesis of the importance of the immunity background in the risk of congenital malaria.

A major limitation of those two very similar meta-analyses, that can be traced back to the original studies pooled, and a major caveat for their adequate interpretation, are the difficulties in linking malaria infection to clinical disease in this particular age group. As previously mentioned, in malaria-endemic areas, attributing to malaria the causality of the symptoms seen in a sick newborn requires a comprehensive and thorough exclusion of other possible causes, otherwise clinical misattribution may be significant. A second important limitation is the major focus on a single continent (Africa) and species (*P. falciparum*), whereby very few studies (and data) have been included from other species and malaria-endemic regions of the world. Having said so, meta-analyses like the ones recently published can address the neglect and broaden the poor visibility that congenital and neonatal malaria have traditionally suffered, on account of their until recently assumed low burden of disease. They can also propose contextual frameworks based on the endemicity characteristics that can help stratify regions and countries in terms of potential risks to health of vertical transmission, thus defining more proactive protocols for malaria investigation in the sick newborn. Indeed, having succeeded on bypassing this first visibility hurdle, it is now time to focus on re-defining what particular strategies are best suited for the prevention and treatment of congenital and neonatal malaria. In this respect, approaches will need to focus both on pregnant mothers (including early diagnosis and prompt treatment, maternal chemoprevention or vaccination) and the newborn, where newborn-friendly formulations and weight-specific adjustments of the dosing of the drugs available to cure malaria will be required, in an effort to resolve some of the unmet needs [16] of this particularly vulnerable–in its own way–group.

## Data Availability

Not applicable.
